# The Protective Effects of Goitrin on LPS-Induced Septic Shock in C57BL/6J Mice via Caspase-11 Non-Canonical Inflammasome Inhibition

**DOI:** 10.3390/molecules28072883

**Published:** 2023-03-23

**Authors:** Deqing Ruan, Jingyi Yang, Qianfei Luo, Yanhong Shi, Lili Ding, Zhengtao Wang, Rui Wang, Li Yang

**Affiliations:** 1 School of Pharmacy, Shanghai University of Traditional Chinese Medicine, Shanghai 201203, China; 2 Shanghai Key Laboratory of Compound Chinese Medicines and The Ministry of Education (MOE) Key Laboratory of Standardization of Chinese Medicines, Institute of Chinese Materia Medica, Shanghai University of Traditional Chinese Medicine, Shanghai 201203, Chinanail8219@126.com (L.D.);; 3 Department of Molecular Pharmacology, Yunnan University of Chinese Medicine, Kunming 650500, China; 4 Institute of Interdisciplinary Integrative Medicine Research, Shanghai University of Traditional Chinese Medicine, Shanghai 201203, China

**Keywords:** goitrin 1, septic shock 2, lipopolysaccharide 3, caspase-11 4, pyroptosis 5

## Abstract

Septic shock is defined as a subset of sepsis, which is associated with a considerably high mortality risk. The caspase-11 non-canonical inflammasome is sensed and activated by intracellular lipopolysaccharide (LPS) leading to pyroptosis, it plays a critical role in septic shock. However, there are few known drugs that can control caspase-11 non-canonical inflammasome activation. We report here that goitrin, an alkaloid from *Radix Isatidis*, shows protective effects in LPS-induced septic shock and significant inhibitory effect in caspase-11 non-canonical inflammasome pathway. Male C57BL/6J were injected intraperitoneally with LPS (20 mg/kg) to induce experimental septic shock. The results demonstrated that the survival rates of mice pretreated with goitrin or Toll-like receptor 4 (TLR4) inhibitor TKA-242 increased, and LPS-induced hypothermia and lung damage improved by inhibiting inflammatory response. Elucidating the detailed mechanism, we surprisingly found goitrin is really different from TAK-242, it independent of the TLR4 signal activation, but significantly inhibited the activation of caspase-11 non-canonical inflammasome, including cleaved caspase-11 and N-terminal fragment of gasdermin D (GSDMD-NT). Furthermore, with a nonlethal dose of the TLR3 agonist poly(I:C)-primed and subsequently challenged with LPS to induce caspase-11-mediated lethal septic shock, the efficacy of goitrin had been verified. Those results revealed the effect of goitrin in protective against LPS-induced septic shock via inhibiting caspase-11 non-canonical inflammasome, which provided a new therapeutic strategy for clinical treatment of septic shock.

## 1. Introduction

Septic shock is a major challenge in intensive care units that develops as life-threatening organ dysfunction resulting from dysregulated host responses to microbial infection. According to the Third International Consensus Definition for Sepsis and Septic Shock [1,2] septic shock is a subset of sepsis. Septic shock is associated with a high risk of death worldwide [3,4]. 

Lipopolysaccharide (LPS) is the main constituent of the outer membrane of Gram-negative bacteria [5,6] and is a key pathogen-associated molecular pattern in septic shock. Seminal studies have shown the host identification of a wide variety of Gram-negative bacteria depends on innate immune surveillance of LPS [7,8]. On the one hand, LPS promotes septic shock via the activation of macrophage cell surface receptor Toll-like receptor 4 (TLR4) [9,10], which leads to the secretion of proinflammatory cytokines including tumor necrosis factor-α (TNF-α), interleukin-6 (IL-6), and interleukin-1β (IL-1β). On the other hand, caspase-11 (also known as caspase-4 in humans) can directly bind to the intracellular LPS [11,12], leading to their oligomerization followed by pyroptosis, also termed the noncanonical inflammasome path way [13]. Specifically, caspase-11 directly promotes pyroptosis through the cleavage of the pore-forming protein gasdermin D (GSDMD) to generate an N-terminal fragment of gasdermin D (GSDMD-N). Subsequently, caspase-11 can trigger a secondary activation of the canonical NLRP3/caspase-1 inflammasome for IL-1β maturation and release [14,15]. Due to the fact that TLR4 inhibitors did not achieve the expectations in clinical trials as anti-sepsis drugs [16,17], LPS-mediated pyroptosis was found to be the main driver for septic shock and underlines the pivotal role of caspase-11 or caspase-4 as an LPS receptor [18,19]. However, there are few reports about drugs that can control caspase-11 non-canonical inflammasome excess activation by LPS.

**Figure 1:**
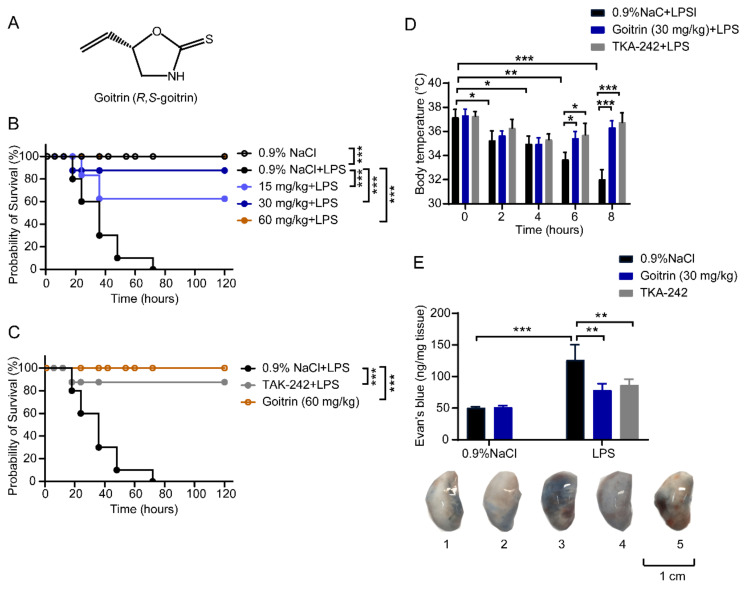
Protective effects of goitrin in LPS-challenged mice. Mice were peritoneally injected with 0.9% NaCl, goitrin (15, 30, or 60 mg/kg) or TAK-242 (3 mg/kg) 1 h before LPS (20 mg/kg) intravenous injection. (**A**) Structure of goitrin. (**B**,**C**) The survival rates were observed and calculated for 5 days (*n* = 10, *** *p* < 0.001, Kaplan–Meier survival analysis). (**D**) Body temperature of mice was monitored by measuring the temperature of the rectum and recorded for 8 h (*n* = 6). (**E**) The lung vessel permeability was measured by intravenous injection of Evans Blue dye after LPS injection for 8 h. “1” for 0.9%NaCl group; “2” for goitrin(30 mg/kg) group; “3” for 0.9%NaCl + LPS (20 mg/kg) group; “4” for TAK-242 + LPS group; “5” for goitrin + LPS group (*n* = 6). Similar results were obtained in at least three independent experiments. The data are presented as the means ± SD. * *p* < 0.05, ** *p* < 0.01, *** *p* < 0.001 (ANOVA LSD test).

Natural products from traditional Chinese medicines (TCMs) constitute a significant portion of the pharmaceutical market today owing to their numerous biological activities. Goitrin (also known as *R*,*S*-goitrin, chemical structure shown in Figure 1A) is an alkaloid from *Radix Isatidis*. Based on our previous studies, it has been used for quality evaluation of *Radix Isatidis* and granules recorded in the Chinese Pharmacopoeia (2020 Edition) [20,21,22]. *Radix Isatidis*, the dried roots of *Isatis indigotica* Fort. (Fam. Brassicaceae), was shown to have a wide range of pharmacological activities, including heat clearing and detoxification, blood cooling, and throat clearing for thousands of years in Asia [23,24]. Moreover, its granules have been occupying a huge share of the international market in recent decades. Previous studies only paid attention to the antiviral and virus-induced inflammatory inhibition activity of goitrin [25,26]. Our recent research has shown that the aqueous extract of *Radix Isatidis* significantly reduced LPS-induced lung damage and inflammation by inhibiting the IFN-β pathway [27]. However, the potential effect of goitrin on septic shock has not been revealed in past studies. In the current study, we provide evidence indicating that goitrin is able to inhibit caspase-11 activation and pyroptosis in LPS-challenged C57BL/6J mice. Survival rates of mice can be improved in both LPS-induced and TLR3 agonist poly(I:C)/LPS-induced septic shock in a dose-dependent manner by pretreatment with Giotrin. Indeed, it inhibits the LPS-induced activation of caspase-11 in lung tissues, leading to reduced NLRP3, caspase-1 activation, and mature IL-1b release, as well as decreased pyroptosis. In conclusion, our results indicate that Goitrin exhibits a protective role against LPS-induced septic shock via caspase-11 non-canonical inflammasome inhibition, which is independent of the TRL4 pathway.

## 2. Results

### 2.1. Protective Effects of Goitrin in LPS-Induced Septic Shock 

Mortalities were used as the experimental endpoint in the LPS-induced septic shock model [12,19], so we investigated the anti-septic shock effects of goitrin using LPS-challenged mice survival rates. As shown in Figure 1B, the normal mice challenged with a lethal dose of LPS were dead within 48 h. However, the survival rates of goitrin-pretreated mice were obviously improved in a dose-dependent manner: the survival rates of mice in low (15 mg/kg), medium (30 mg/kg), and high (60 mg/kg) dose groups of Goitrin were 60%, 90%, and 100%, respectively (Figure 1B). Meanwhile, the survival rate of the TLR4 inhibitor TAK-242 (3 mg/kg) positive group was 90% (Figure 1C). In addition, the mice administered with a high dose of goitrin (60 mg/kg) had good survival conditions (Figure 1C). 

LPS-induced hypothermia is also a well-known surrogate for the severity of systemic inflammation and illness in mice which has been extensively used as an endpoint for the LPS-induced septic shock model [28]. We measured the rectal temperature in the mice with LPS-induced septic shock. Hypothermia was significantly induced at 2 h after LPS (20 mg/kg) administration and continued to decrease at 4, 6, and 8 h. As expected, hypothermia was significantly inhibited by goitrin (30 mg/kg) or TAK-242 (3 mg/kg) (Figure 1D).

**Figure 2:**
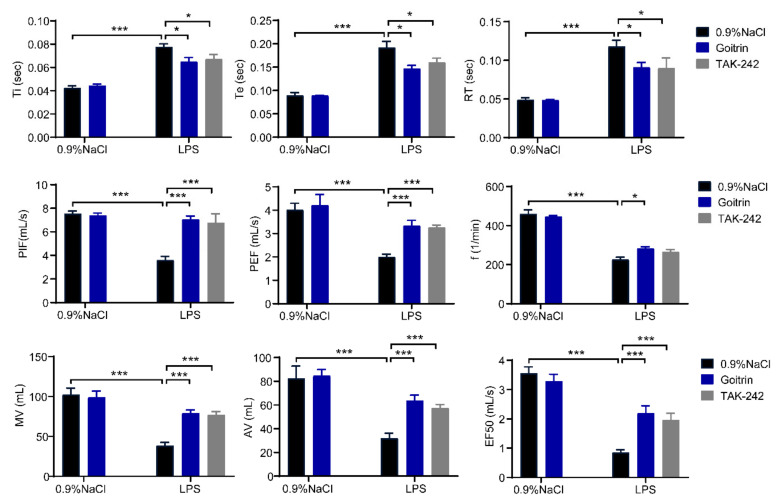
Goitrin improved lung function and airway response in LPS-induced mice. Mice were peritoneally injected with 0.9% NaCl, goitrin (30 mg/kg), or TAK-242 (3 mg/kg) 1 h before LPS (20 mg/kg) intravenous injection. After 8 h exposure to LPS, the respiratory parameters were measured by whole-body plethysmography (WBP) (*n* = 6). Similar results were obtained in at least three independent experiments. The data are presented as the means ± SD. * *p* < 0.05, *** *p* < 0.001 (ANOVA LSD test).

**Figure 3:**
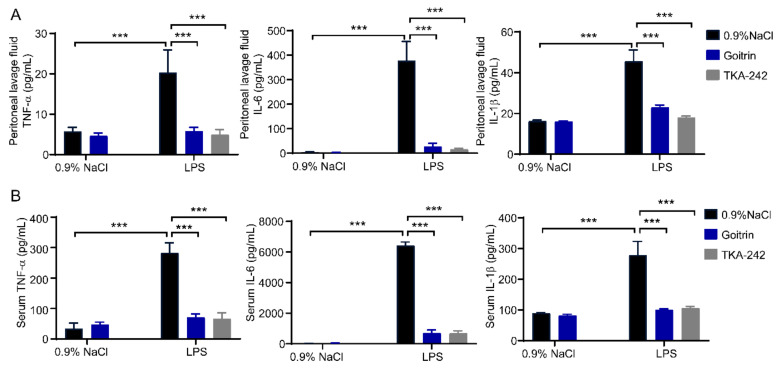
Goitrin inhibited IL-6, IL-1β, and TNF-α in LPS-challenged mice. Mice were peritoneally injected with 0.9% NaCl, goitrin (30 mg/kg), or TAK-242 (3 mg/kg) 1 h before LPS (20 mg/kg) intravenous injection. Protein concentrations of IL-6, IL-1β, and TNF-α in peritoneal lavage fluid (**A**) and serum (**B**) were measured after LPS injection for 8 h (*n* = 6). Similar results were obtained in at least three independent experiments. The data are presented as the means ± SD. *** *p* < 0.001 (ANOVA LSD test).

Specfically because the lungs are the most vulnerable and critical organ during septic shock, we measured lung vessel permeability by intravenous injection of Evans Blue dye to reflect the damage degree of the vascular endothelial barrier in the lung tissues of mice [29,30]. As shown in Figure 1E, the accumulation of Evans Blue dye in lung tissues of mice induced by LPS (20 mg/kg) was obviously increased after LPS injection for 8 h (Figure 1E-3). As we expected, goitrin (30 mg/kg) or TAK-242 (3 mg/kg) reduced the accumulation of Evans Blue dye in the lung tissues, showing that the mice were protected from lung damage.

The whole-body plethysmograph (WBP) system was used to monitor lung function and airway response in mice [31]. As shown in Figure 2, time of inspiration (Ti), time of expiration (Te) and relaxation time (RT) were significantly increased in the LPS challenged for 8 h, meanwhile the breathing frequency (f), peak inspiratory flow (PIF), peak expiratory flow (PEF), minute ventilation (Mv), accumulative volume (AV), and flow rate at 50% expiratory volume (EF50) were decreased. In keeping with our results above, goitrin (30 mg/kg) or TAK-242 (3 mg/kg) significantly improved the lung function and airway response of LPS-induced septic shock mice (Figure 2).

### 2.2. Goitrin Alleviates LPS-Induced Systemic Inflammation

To assess the effects of goitrin in LPS-induced systemic inflammation, we detected the levels of pro-inflammatory cytokines in peritoneal lavage fluid and serum. Our results shown that compared with the 0.9% NaCl mice group, LPS (20 mg/kg) dramatically raised the levels of pro-inflammatory cytokines after 8 h in the peritoneal lavage fluid, including IL-6, IL-1β and TNF-α. While treatment with goitrin (30 mg/kg) or TAK-242 (3 mg/kg) clearly reduced the protein levels of pro-inflammatory cytokines (Figure 3A). Consistent with the results in peritoneal lavage fluid, we obtained the same results in serum, goitrin (30 mg/kg) or TAK-242 (3 mg/kg) clearly reduced the protein levels of IL-6, IL-1β and TNF-α (Figure 3B).

**Figure 4:**
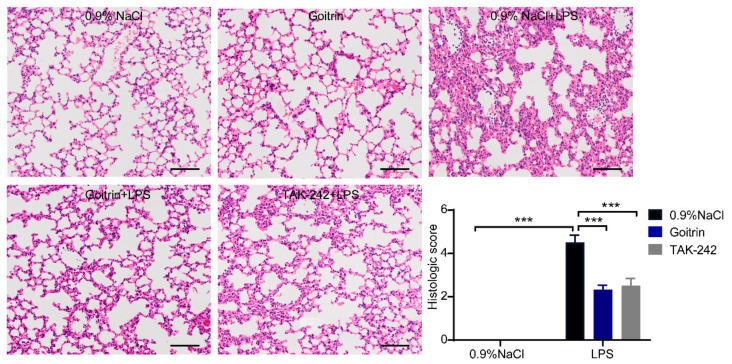
Goitrin reduced lung injury and inflammatory cell infiltration. Mice were peritoneally injected with 0.9% NaCl, goitrin (30 mg/kg), or TAK-242 (3 mg/kg) 1 h before LPS (20 mg/kg) intravenous injection (*n* = 6). Pathological analysis of the mice livers and lungs after LPS injection for 8 h was determined using hematoxylin and eosin staining (scale bar = 50 μm). Then, lung tissue injury was scored by a blinded assessment method for five categories. The data are presented as the means ± SD. *** *p* < 0.001 (ANOVA LSD test).

Lung is the most common severe injury organ caused by sepsis. Inflammatory cytokines play a key role in the process of acute lung injury [32,33]. Histological changes in the lungs were examined by H and E staining to measure the inflammatory response in the lungs. Lung tissues from the 0.9%NaCl group mice displayed normal alveolar walls and no inflammatory cell infiltration. In comparison, the LPS-induced mice showed obvious alveolar wall thickening with profound inflammatory cell infiltration (Figure 4). In contrast, the treatment of goitrin (30 mg/kg) or TAK-242 (3 mg/kg) significantly reduced lung injury and inflammatory cell infiltration. Consistent with the above results, LPS-induced mice had significantly higher lung injury scores than the 0.9%NaCl group mice. Whereas, the lung injury score of the goitrin or TAK-242 group mice were significantly lower those of of the LPS group.

**Figure 5:**
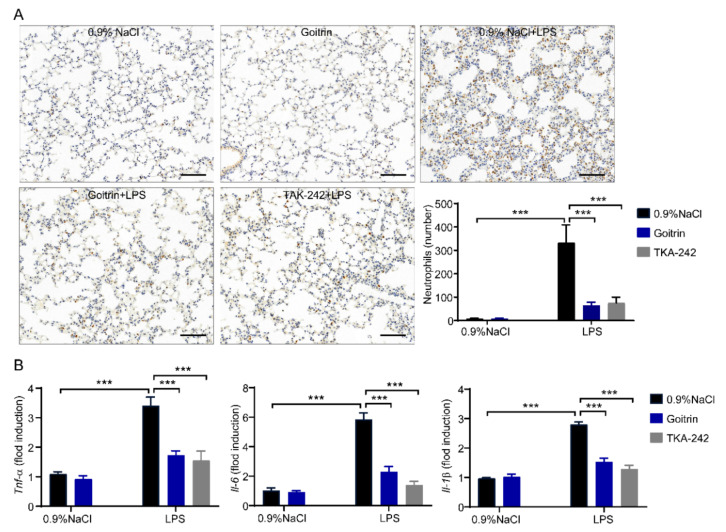
Goitrin attenuated LPS-induced inflammatory responses in lung. Mice were peritoneally injected with 0.9% NaCl, goitrin (30 mg/kg), or TAK-242 (3 mg/kg) 1 h before LPS (20 mg/kg) intravenous injection (*n* = 6). The accumulation of neutrophils was quantified by IHC staining (**A**) and mRNAs expression (**B**) were measured in the lung after LPS injection for 8 h. The data are presented as the means ± SD. *** *p* < 0.001 (ANOVA LSD test). (scale bar = 50 μm).

Next, we checked the accumulation of inflammatory cells by staining MPO (myeloperoxidase, a neutrophil marker), which is commonly used to assess the quantification of neutrophil accumulation in tissues. As shown in Figure 5A, neutrophils were significantly reduced in goitrin (30 mg/kg) or TAK-242 (3 mg/kg) pretreated groups after LPS-induced for 8 h. Moreover, we investigated proinflammatory cytokines expression including *Il-1β*, *Il-6* and Tnf-α, the expression of those mRNA was significantly reduced in goitrin (30 mg/kg) or TAK-242 (3 mg/kg) pretreated mice compared with LPS-challenged mice (Figure 5B). Taken together, goitrin is similar to TAK-242 in that it has protective effects against mice septic death and alleviates LPS-induced systemic inflammation.

### 2.3. Goitrin Does Not Affect the LPS-Activated TRL4 Receptor

**Figure 6:**
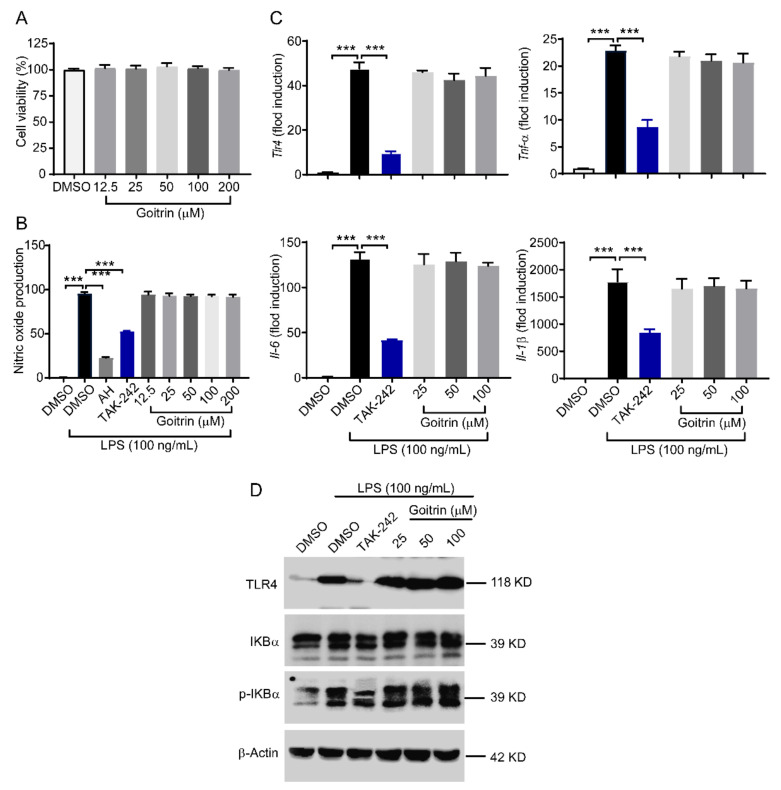
Effects of goitrin on the activation of TRL4 in LPS-stimulated RAW 264.7 cells. (**A**) CCK-8 assay. RAW264.7 cells were treated with goitrin at different concentrations (12.5, 25, 50, 100, or 200 µM) for 24 h (*n* = 6). (**B**) Determination of NO production. RAW264.7 cells were pretreated with goitrin (25, 50, or 100 µM), AH (50 μM), or TAK-242 (100 nM) for 30 min before adding LPS (100 ng/mL). After incubation for 24 h, the culture supernatant and cells were collected for analysis (*n* = 6). (**C**) The mRNA expression of *Tlr4* and pro-inflammatory cytokines. RAW264.7 cells were pretreated with goitrin (25, 50, or 100 µM) or TAK-242 (100 nM) for 30 min before adding LPS (100 ng/mL). After incubation for 4 h, the culture supernatant and cells were collected for analysis (*n* = 6). (**D**) Measurement of the protein of TRL4 and phosphorylation of IKBα. RAW264.7 cells were pretreated with goitrin (25, 50, or 100 µM) or TAK-242 (100 nM) for 30 min before adding LPS (100 ng/mL). After incubation for 30 min, the cells were collected for analysis (*n* = 3). Data represent the mean ± SD of three independent experiments. The data are presented as the means ± SD. *** *p* < 0.001 (ANOVA LSD test).

Our result clearly showed that goitrin and TAK-242 had a similar anti-septic shock effect. However, is goitrin similar to TAK-242 that can inhibit the LPS-activated TRL4 receptor? To confirm the effects of goitrin to elucidate the underlying molecular mechanisms, the mouse macrophage cell line RAW264.7 was used. The results of the CCK-8 assay showed that goitrin had no toxic effect on RAW264.7 cell proliferation at the concentration of 12.5~200 μM (Figure 6A). RAW264.7 cells were pretreated with goitrin for 30 min and then stimulated with LPS (100 ng/mL). The exposure activated TRL4-dependent RAW264.7 cell inflammation reflection. We first measured nitric oxide (NO) secretion in the supernatants, which has an important role in mediating cellular immune inflammation, and it was significantly enhanced after LPS stimulation for 24 h (Figure 6B). As expected, nitric oxide synthase inhibitor aminoguanidine hydrochloride (AH, 50 μM) or TAK-242 (100 nM) was more potent to inhibit LPS-induced NO production. Unfortunately, treatment with Goitrin at different concentrations (12.5, 25, 50, 100, and 200 µM) had little affected on NO production (Figure 6B). Similarly, LPS stimulation significantly upregulated the mRNA expression of *Tlr4* and pro-inflammatory cytokines *Tnf-α*, *Il-6*, and *Il-1β*. Treatment with goitrin at different concentrations (12.5, 25, 50, and 100 µM) hardly inhibited the expression of their mRNA expression. Unlike goitrin, TAK-242 (100 nM) strongly inhibited the activation of TRL4 by LPS (Figure 6C).

Furthermore, to examine the effect of goitrin on TRL4 transcription, a Western blot was used to analyze the TRL4 and phosphorylation of IKBα downstream kinases of TRL4. Upon LPS stimulation, TRL4 was expressed and the phosphorylation of IKBα was induced and TAK-242 inhibited the above reaction. However, goitrin had little impact (Figure 6D). These results clearly indicate that goitrin does not affect the LPS-activated TRL4 receptor, that is to say, the anti-septic shock effect of goitrin is different from the TAK-242.

### 2.4. Goitrin Inhibits Caspase-11 Non-Canonical Inflammasome Activation

**Figure 7:**
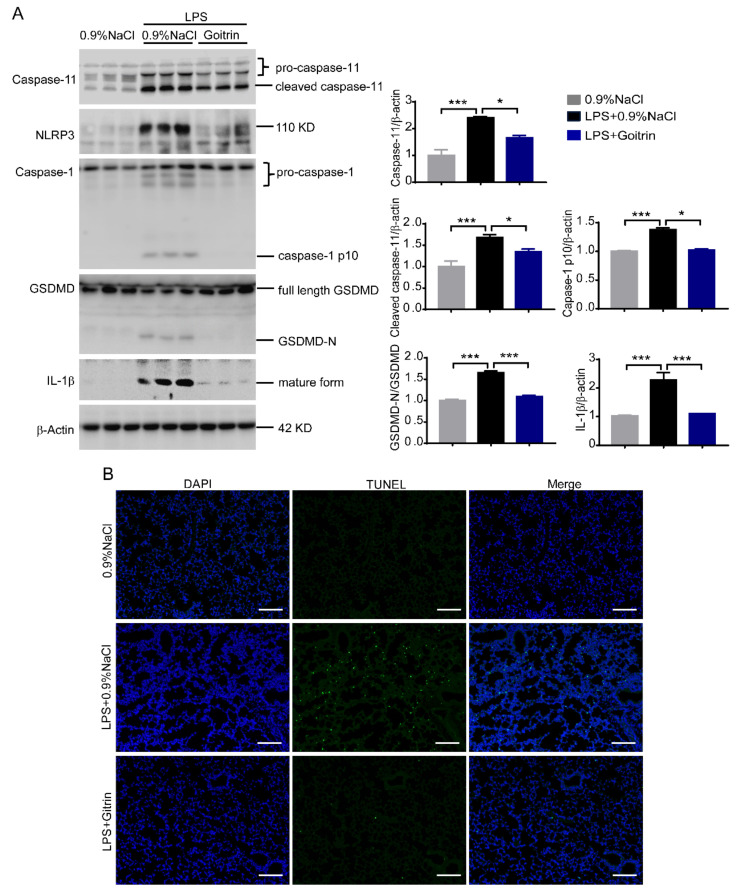
Goitrin prevented the activation of the caspase-11/GSDMD signal and pyroptosis. Mice were peritoneally injected with 0.9% NaCl or goitrin (30 mg/kg) 1 h before LPS (20 mg/kg) intravenous injection and the mice were sacrificed 8 h later. (**A**) Protein levels of caspase-11, cleaved caspase-11, cleaved GSDMD (GSDMD-N), NLRP3, caspase-1, and mature IL-1β were analyzed in lung tissues (*n* = 3). (**B**) DNA breakage was assayed by the TUNEL method in lung tissues (*n* = 6). The data are presented as the means ± SD. * *p* < 0.05, *** *p* < 0.001 (ANOVA LSD test). (scale bar = 50 μm).

As we pointed out in the background, cytoplasmic LPS can directly bind to and activate caspase-11 independently of TLR4, and this mechanism may be more critical to driver septic shock than the recognition of LPS by TLR4. It was therefore necessary to ascertain whether goitrin could prevent the activation of the caspase-11/GSDMD signal, which was reported as a marker of pyroptosis induced by LPS. We first checked their expression in mice lung tissues. As shown in Figure 7A, protein levels of cleaved caspase-11 and cleaved GSDMD (GSDMD-N) were all enhanced in mice lung tissues after challenged LPS for 8 h. However, it is surprising that pretreatment with goitrin could reduce the protein levels of pro-caspase11, cleaved caspase-11, and cleaved GSDMD (GSDMD-N) in mice lung tissue. Meanwhile, we measured the activity of NLRP3, caspase-1, and the production of mature IL-1β in these lung tissues. Compared to the 0.9% NaCl mice group, the activity of NLRP3, cleaved caspase-1 (caspase-1 p10), and mature IL-1β were all significantly increased in the LPS-challenged mice group, consistent with the caspase-11/GSDMD expression. Indeed, the levels of these proteins were also significantly decreased in the goitrin pretreated mice group (Figure 7A).

Furthermore, pyroptosis mediated by caspase-11 is a programmed cell death process, which undergo extensive DNA breakage and degradation. In order to detect the DNA breakage in lung tissues, the terminal deoxynucleotidyl transferase (TdT)-mediated dUTP nick-end labelling (TUNEL) technique has been used. It was consistent with above results, the number of TUNEL staining positive cells in lung tissues was significantly increased in LPS-challenged mice, while goitrin could significantly reduce the number of TUNEL staining positive cells (Figure 7B). All the above results clearly demonstrated that Giotirn significantly inhibited the activation of caspase-11 non-canonical inflammasome activation to against LPS-induced septic shock, but not effects of TRL4 activation.

### 2.5. Goitrin Attenuates Caspase-11-Mediated Lethal Septic Shock

**Figure 8:**
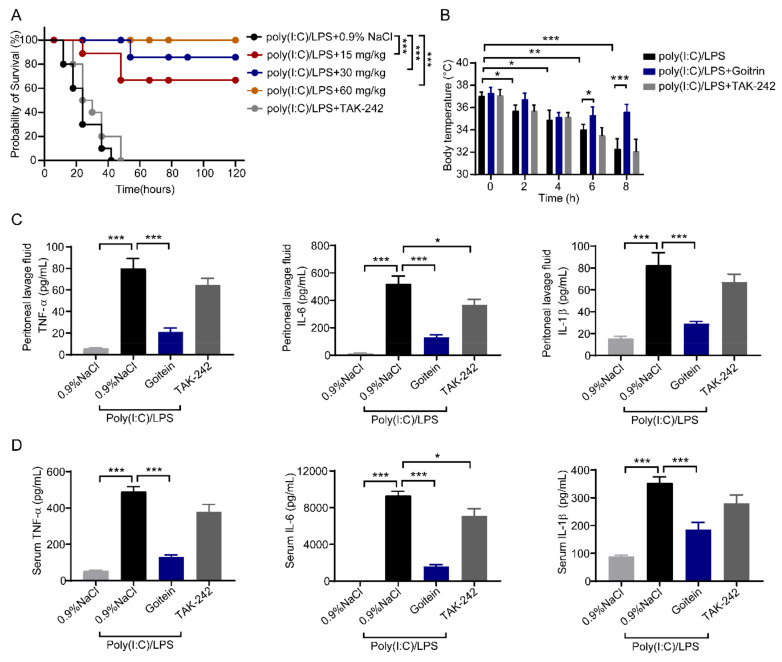
Goitrin attenuates caspase-11-mediated lethal septic shock. (**A**) Kaplan–Meier survival analysis and body temperature were monitored. Mice were primed with poly(I:C) [4 mg/kg, ip (intraperitoneally)] and challenged 6 h (h) later with LPS (20 mg/kg, ip) in the absence or presence of goitrin (15, 30, or 60 mg/kg) or TAK-242 (3 mg/kg) (*n* = 10, *** *p* < 0.001, Kaplan–Meier survival analysis). (**B**) Body temperature of mice was monitored by measuring the temperature of the rectum and recorded for 8 h (*n* = 6). (**C**,**D**) Quantitation of IL-6, IL-1β, and TNF-α levels in peritoneal lavage fluid and serum. Mice were primed with poly(I:C) [4 mg/kg, ip (intraperitoneally)] and challenged 6 h (h) later with LPS (20 mg/kg, ip) in the absence or presence of goitrin (30 mg/kg) or TAK-242 (3 mg/kg), the mice were sacrificed 8 h later (*n* = 6). * *p* < 0.05, ** *p* < 0.01, *** *p* < 0.001 (ANOVA LSD test).

Based on the above results, we next investigated whether goitrin attenuates caspase-11-mediated lethal septic shock independently of TLR4 in vivo. We circumvented the TLR4 priming issue by intraperitoneal injection of the mice with a nonlethal dose of the TLR3 agonist poly(I:C) and subsequently challenged them with LPS to induce caspase-11-mediated lethal septic shock as reported [11,14,34]. Consistent with previous reports [11,14], TAK-242 (3 mg/kg) showed no resistance to poly(I:C)-primed cytosolic LPS-induced septic shock (Figure 8A), supporting the standpoint that LPS-induced septic shock requires activation of the caspase-11 non-canonical inflammasome but not TLR4 [35]. In contrast, administration of Goitrin rescued poly(I:C)/LPS-induced septic shock in dose-dependently, the survival rates of mice in low (15 mg/kg), medium (30 mg/kg) and high (60 mg/kg) dose groups of Goitrin were 70%, 90% and 100%, respectively (Figure 8A). Next, we also measured the rectal temperature, Goitrin (30 mg/kg) significantly inhibited poly(I:C)/LPS-mediated hypothermia at 6 h and 8 h points (Figure 8B). After that, the release of TNF-α, IL-6, and IL-1β proteins was also detected in mice peritoneal lavage fluid and serum. Consistent with LPS-induced mice, Goitrin (30 mg/kg) had an obvious effect in the production of these cytokines in poly(I:C)/LPS-mediated mice. Meanwhile, TAK-242 (3 mg/kg) only inhibited IL-6 (Figure 8C,D).

**Figure 9:**
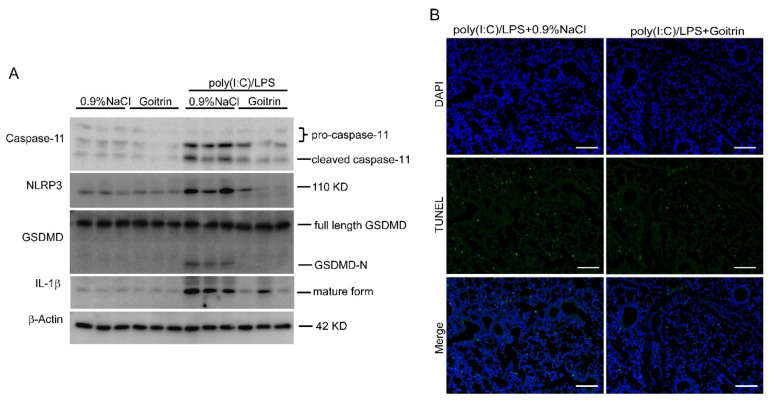
Goitrin inhibited caspase-11 non-canonical inflammasome activation poly(I:C)/LPS-mediated mice. Mice were primed with poly(I:C) [4 mg/kg, ip (intraperitoneally)] and challenged 6 h (h) later with LPS (20 mg/kg, ip) in the absence or presence of goitrin (30 mg/kg) or TAK-242 (3 mg/kg), the mice were sacrificed 8 h later (*n* = 6). (**A**) Protein levels of caspase-11, cleaved caspase-11, cleaved GSDMD (GSDMD-N), NLRP3, and mature IL-1β were analyzed in lung tissues (*n* = 3). (**B**) DNA breakage was assayed by the TUNEL method in lung tissues (*n* = 6). (scale bar = 50 μm).

In order to further elucidate the caspase-11 non-canonical inflammasome activation, here we again measured the protein level of caspase-11/GSDMD signal in mice lung tissues. In line with biochemical results, the Western blot showed that protein levels of cleaved caspase-11, cleaved GSDMD (GSDMD-N), NLRP3, and mature IL-1β were enhanced in mice lung tissues after challenging by poly(I:C)/LPS for 8 h (Figure 9A). As we expected, among the lung tissues of mice treated with goitrin, not only were cleaved caspase-11, cleaved GSDMD (GSDMD-N), and mature IL-1β markedly decreased, but NLRP3 was also inhibited (Figure 9A). Moreover, in our previous study DNA breakage was prevented by pretreating with goitrin in lung tissues (Figure 9B). These results further confirmed that goitrin protected against septic shock by inhibiting caspase-11 non-canonical inflammasome activation and was independent of TLR4 in mice.

## 3. Discussion

It has long been believed that the robust TLR4 plays a critical role in the pathogenesis of septic shock and sepsis, which mainly regulate the infiltration of inflammatory cells and the production of pro-inflammatory cytokines [36,37]. Although blocking TLR4 can result in the protection of wild-type mice from lethal endotoxemia and lethal E. coli induced sepsis, TLR4 inhibitors and inflammatory cytokine antagonists do not meet the expectations in clinical trials as anti-sepsis drugs [38,39]. Here, we showed that goitrin inhibited LPS-induced lethal septic shock, hypothermia, and systemic inflammation in mice, which had a similar effect with TAK-242. However, we showed lines of results that goitrin had of no effect on the expression of TLR4, phosphorylation of IKBα, or on pro-inflammatory cytokines *Tnf-α*, *Il-6*, and *Il-1β* in LPS-stimulated macrophages.

Caspases are ancient proteases that are essential to basic cellular physiology. Although some caspases are involved in apoptosis, the inflammatory caspases-1 and caspase-11 cause pyroptosis, a type of lytic programmed cell death. Caspase-1 is activated by canonical inflammasomes, which signal through the adaptor ASC. NLRC4 and NLRP3 can also directly activate caspase-1 [40,41]. Caspase-11, in contrast to caspase-1, is activated without the involvement of any known conventional inflammasome pathways and has been termed the non-canonical inflammasome [12]. The innate immune system detects Gram-negative bacteria partly by recognizing LPS [42]. An innovative method of recognizing LPS was uncovered via extensive investigation [7,43]. This way of surveillance for intracellular LPS by caspase-11 in mice and caspase-4 in humans causes pyroptosis in a TLR4-independent manner via the pore-forming protein GSDMD, which inadvertently causes the maturation of IL-1β in some cell types [44]. Studies have revealed that the presence of the caspase-11 non-canonical inflammasome sensing pathway is not only relevant to prepyrosis of immune cells but also to sepsis pathogenesis as well [45,46]. During the late stage of sepsis, patients can suffer from severe immunosuppression due to a higher rate of lymphocyte apoptosis and tolerance [47,48]. Thus, LPS-induced pyroptosis via inflammasome activation might contribute to immunosuppression in later stages of sepsis as well as to organ dysfunction [49,50]. 

Given the critical role of intracellular LPS sensing in the development of septic shock, inhibiting the activation of caspase-11/4 and subsequent effector pathways has important clinical ramifications. Targeting both caspase-11 and GSDMD is considered a potential therapeutic avenue for the treatment of septic shock and sepsis. While remarkable progress has been made in this area, there are still only a few drugs that can inhibit caspase-11 non-canonical inflammasome. Despite the fact that several of the caspases-4 small molecule inhibitors and GSDMD pore-formation inhibitors recently identified in studies have received FDA medication approval [7,51,52], they share many of the same negative effects as nonsteroidal anti-inflammatory drugs. It is most noteworthy that in the absence of caspase-11, mice develop an acute susceptibility to infection from bacteria such *Burkholderia pseudomallei* and *B. thailandensis* that reproduce in the cytosol after escaping the phagosome [53]. Here, we report that goitrin could not only inhibit protein levels of pro-caspase11, cleaved caspase-11, and cleaved GSDMD (GSDMD-N), but also suppress the noncanonical NLRP3/caspase-1 inflammasome activation in LPS-challenged mice. Notably, caspase-11-dependent cleavage of GSDMD is discussed as a link between LPS-induced activation of caspases and pyroptosis or NLRP3 inflammasome activation, in other words, caspase-11 detection of intracellular LPS mediates noncanonical activation of the NLRP3 inflammasome [54]. These results may add evidence regarding the mechanism of action accounting for previous findings that goitrin inhibited caspase-11/GSDMD signal to ameliorate systemic inflammatory responses and increase the survival of mice with LPS-induced septic shock.

The caspase-11 pathway is inactive unless macrophages are pre-stimulated (primed) with LPS, poly(I:C), IFN-β, or IFN-γ, which likely induces multiple components of the non-canonical inflammasome pathway [53,55,56]. LPS and TLR3 agonist poly(I:C) prime via TLR4 and TLR3, respectively. Poly(I:C) could be used as a priming agent instead of LPS to separate the priming and activation stimuli of caspase-11. The other important finding from our study is that goitrin can also improve the survival rate of mice in poly(I:C)/LPS-induced caspase-11-mediated lethal septic shock implications in a TLR4 independent manner. 

In conclusion, our data indicate that the natural alkaloid goitrin isolated from medicinal herbals is a potential drug for inhibiting caspase-11 activation. Despite the critical role of caspase-11-induced pyroptosis in septic pathogenesis, few caspase-11 inhibitors have been found yet: goitrin represents a useful candidate for further research and for the development of drugs against bacterial sepsis-related diseases. Traditional Chinese medicine has been used to treat chronic diseases in the past, and although detailed studies are required, our study elucidates the effects of traditional Chinese medicine on acute illness.

## 4. Materials and Methods 

### 4.1. Chemicals and Reagents

Goitrin (purity > 98%) was provided by the Shanghai R and D Centre for Standardization of Traditional Chinese Medicines (Shanghai, China). Lipopolysaccharides (LPS, Escherichia coli O111:B4) and Aminoguanidine Hydrochloride (AH) were obtained from Sigma (St. Louis, MO, USA). TAK-242 was obtained from MedChemExpress. Polyinosinic-polycytidylic acid [poly(I:C) LMW] was obtained from InvivoGen (Toulouse, France).

### 4.2. Animals and Experimental Establishment

Male C57BL/6J mice (age, 6–8 weeks, and weight, 18–22 g), were obtained from the Shanghai SLAC Laboratory Animal Co., Ltd. (Shanghai, China). All efforts were made to minimize animal suffering and to reduce the number of animals used in the experiments. Animal experiments were conducted in accordance with the “Regulations on the Administration of Laboratory Animals” promulgated by the Ministry of Science and Technology of the People’s Republic of China (1988) No. 134 and approved by the Animal Ethics Committees of Shanghai University of Traditional Chinese Medicine. Animals were housed at a constant room temperature (25 °C) and relative humidity (70%) with a 12 h light/12 h dark cycle. Food and tap water were available ad libitum.

For LPS-induced septic shock, mice were randomly divided into four groups: 0.9%NaCl, LPS(20 mg/kg), TAK-242(3 mg/kg) + LPS, and goitrin (15, 30, or 60 mg/kg) + LPS groups, respectively. In the goitrin (15, 30, or 60 mg/kg) or TAK-242 (3 mg/kg) pretreated sepsis groups, mice were pretreated with goitrin or TAK-242 through intraperitoneal injection for 1 h and then injected intravenously with 20 mg/kg LPS to induce experimental sepsis. Mice in the LPS group were administered with equivalent volumes of 0.9% NaCl (vehicle) for 1 h before LPS injection. In the 0.9% NaCl group, mice were only administered with an equivalent volume of 0.9% NaCl.

As previously described [14,34], for poly(I:C)/LPS-induced caspase-11-mediated lethal septic shock mice, mice were primed with poly(I:C) [4 mg/kg, ip (intraperitoneally)] for 6 h and then intraperitoneally injected with different concentrations of goitrin (15, 30, or 60 mg/kg), TAK-242 (3 mg/kg), or 0.9% NaClat 1 h before being challenged with LPS (20 mg/kg, ip).

### 4.3. Survival Analysis and Measurement of Body Temperature 

For survival analysis, general conditions and mouse mortality were observed up to 5 days. For body temperature measurement, we monitored rectal temperature for 8 h. The body temperature of mice was measured once per hour by using a human electronic thermometer; the thermometer was gently inserted 1.0–1.5 cm into the rectum using petroleum jelly. 

### 4.4. Cell Culture and Stimulation

Murine macrophage RAW 264.7 cells were purchased from the Cell Bank of the Chinese Academy of Sciences (Shanghai, China) and cultured in Dulbecco’s modified Eagle’s medium (Thermo Fisher Scientific, Waltham, MD, USA) supplemented with 10% FBS (Thermo Fisher Scientific, Waltham, MD, USA), 100 U/mL of penicillin (Thermo Fisher Scientific, Waltham, MD, USA), and 100 g/mL of streptomycin (Thermo Fisher Scientific, Waltham, MD, USA) at 37 °C with 5% CO_2_. The RAW 264.7 cells were plated at a density of 5 × 10^5^ cells/mL in 6-well polystyrene plates (Eppendorf, Hamburg, Germany) and pre-incubated for 24 h. Then, the cells were pre-treated with goitrin, AH (50 μM), or TAK-242 (100 nM) for 30 min before the addition of 100 ng/mL LPS.

### 4.5. Measurement of Lung Vessel Permeability

Lung vessel permeability was measured as previously described [30,57]. Briefly, mice were placed in a cage under the heat lamp for 5~10 min in order to dilate the blood vessels. Slowly, 200 μL of dye was injected into the tail vein of the mice. Then, the mice back were returned to their cages and observed for 45 min. For anesthesia, 1% sodium pentobarbital was used. Each mouse was weighed and intraperitoneally injected according to 10 μL/g body weight. The needle with 1×PBS was carefully inserted into the left ventricle, and the right ventricle was opened to drain the fluid. Slowly but continuously, 1×PBS was poured into the heart for 5 min. Then, the lungs were carefully dissected from the thoracic cavity and the lung samples weighed. The isolated lung tissue was placed in a centrifuge tube containing 250 μL of formamide by tissue homogenate. It was then incubated at 55 °C in a heating block for 48 h. Lung tissue was centrifuged at 3000× *g* at 4 °C for 15 min. Supernatants were collected and transferred to a new tube. Aliquots of 100 μL/well of lung tissue extract were loaded on a 96-well plate, the absorbance of samples were measured, and standards at 620 nm and 740 nm were measured using a microplate reader. The A620 readings for turbidity in lung tissue were corrected using the correction factor y = 1.193x + 0.007 where x is A740 and A620 corrected = yA620. Standards were used to obtain absolute Evans Blue concentrations in μg/mg.

### 4.6. Lung Function Test

Lung function was tested by whole-body plethysmograph (WBP-4MR, TOW, Shanghai, China). The mice were placed in the animal chamber, and the plethysmography was then conducted based on the measurements of the selected parameter values for 5 min. The time of inspiration (Ti), time of expiration (Te) and relaxation time (RT), frequency (f), peak inspiratory flow (PIF), peak expiratory flow (PEF), minute ventilation (Mv), accumulative volume (AV), and flow rate at 50% expiratory volume (EF50) were determined by the software (ResMass 1.4.2., TOW, Shanghai, China).

### 4.7. Histological and Immunohistochemistry Analysis and the TUNEL Assay

The lungs were collected and fixed with 4% formaldehyde and then embedded with paraffin. Serial 5-μm-thick sections were cut using a microtome (Leica, Heidelberg, Germany) and then stained with hematoxylin and eosin. Light microscopy photographs were taken using Olympus DX45 (Tokyo, Japan). The histological severity of the lung injury was evaluated by Suzuki’s criteria [58]. For immunohistochemistry (IHC) analyses, lung sections incubated with primary antibodies against MPO (Cell Signaling, Boston, MA, USA) overnight at 4 °C, followed by horseradish peroxidase-conjugated secondary antibodies and observe immunoreactive cells with DAB under the microscope. For each stained section, between three and six images from random fields were taken, and at least three mice per group were subjected to each experiment. Image-Pro Plus 6.0 was used for the image analysis of sections.

For the TUNEL assay, lung sections were stained using a TUNEL Cell Apoptosis Detection Kit (Wuhan servicebio technology CO., LTD, Wuhan, Chain) according to the manufacturer’s protocols. The nucleus is blue due to labeling with DAPI. The tunel assay kit is labeled with FITC. Positive apoptosis cells are green.

### 4.8. Quantitative Real-Time Polymerase Chain Reaction Assay

Total RNAs from different experimental groups were extracted using an RNA Faster200 reagent (Fastagen, Shanghai, China) according to the manufacturer’s instructions. RNA was reverse transcribed to generate first strand cDNAs by using the PrimeScript™ RT Master Mix Kit (Takara Bio, Shiga, Japan). The reaction conditions were 37 °C for 15 min and 85 °C for 5 s. The RT-PCR analysis was performed using an SYBR Premix Ex Taq™ (Tli RNaseH Plus) Kit (Takara Bio, Shiga, Japan) according to the manufacturer’s instructions. All the reactions were performed on a 6000 Real-Time PCR System (Thermo Fisher Scientific, Waltham, MD, USA), and the relative quantity was quantified using the 2^−ΔΔCt^ method. The cycle threshold values of the target genes were normalized to that of glyceraldehyde 3-phosphate dehydrogenase (Gapdh) from the same sample.

The primers used for RT-PCR analysis were:

*Gapdh* forward: 5′- GGCCGAGAATGGGAAGCTTGT 

*Gapdh* reverse: 5′- ACATACTCAGCACCGGCCTCA

*Il-1β* forward: 5′- TTAGTCCTCGGCCAAGACAG

*Il-1β* reverse: 5′- GGCAAGGAGGAAAACACAGG

*Tnf-α* forward: 5′- TTCTATGGCCCAGACCCTCA 

*Tnf-α* reverse: 5′- CTCCAAAGTAGACCTGCCCG

*Il-6* forward: 5′- GGGACTGATGCTGGTGACAA

*Il-6* reverse: 5′- ACAGGTCTGTTGGGAGTGGT

### 4.9. Enzyme-Linked Immunosorbent Assay

Blood samples and peritoneal lavage fluid were centrifuged at 3500 rpm for 15 min. TNF-α, IL-1β, and IL-6 concentrations were measured using ELISA kits (Neo Bioscience Technology, Shenzhen, China) according to the manufacturers’ instructions.

### 4.10. Western Blot Analysis

Western blotting analysis was performed as we previously described [27]. Primary antibodies against mouse caspase-11, caspase-1, Gsdmd, IL-1β (Abcam Cambridge, MA, USA), NLR3, TLR4, IKBα, and p- IKBα (Cell Signaling, Boston, MA, USA) were used for these specific proteins and β-actin (Sigma-Aldrich St. Louis, MO, USA) was used as the loading control. 

### 4.11. Statistical Analysis

Statistical analysis was performed using GraphPad Prism (version 7; GraphPad, La Jolla, CA, USA). All the assay values are given as means ± SD. Differences among multiple groups were analyzed using a one-way analysis of variance, followed by the Tukey post hoc test. *p* < 0.05 was considered statistically significant.

## Data Availability

Not applicable.

## Sample Availability

Samples of the Goitrin are available from the authors.
